# The formation of cysteine-linked dimers of BST-2/tetherin is important for inhibition of HIV-1 virus release but not for sensitivity to Vpu

**DOI:** 10.1186/1742-4690-6-80

**Published:** 2009-09-08

**Authors:** Amy J Andrew, Eri Miyagi, Sandra Kao, Klaus Strebel

**Affiliations:** 1 Laboratory of Molecular Microbiology, Viral Biochemistry Section, National Institute of Allergy and Infectious Diseases, NIH, Bethesda, Maryland, 20892-0460, USA

## Abstract

**Background:**

The Human Immunodeficiency virus type 1 (HIV-1) Vpu protein enhances virus release from infected cells and induces proteasomal degradation of CD4. Recent work identified BST-2/CD317 as a host factor that inhibits HIV-1 virus release in a Vpu sensitive manner. A current working model proposes that BST-2 inhibits virus release by tethering viral particles to the cell surface thereby triggering their subsequent endocytosis.

**Results:**

Here we defined structural properties of BST-2 required for inhibition of virus release and for sensitivity to Vpu. We found that BST-2 is modified by N-linked glycosylation at two sites in the extracellular domain. However, N-linked glycosylation was not important for inhibition of HIV-1 virus release nor did it affect surface expression or sensitivity to Vpu. Rodent BST-2 was previously found to form cysteine-linked dimers. Analysis of single, double, or triple cysteine mutants revealed that any one of three cysteine residues present in the BST-2 extracellular domain was sufficient for BST-2 dimerization, for inhibition of virus release, and sensitivity to Vpu. In contrast, BST-2 lacking all three cysteines in its ectodomain was unable to inhibit release of wild type or Vpu-deficient HIV-1 virions. This defect was not caused by a gross defect in BST-2 trafficking as the mutant protein was expressed at the cell surface of transfected 293T cells and was down-modulated by Vpu similar to wild type BST-2.

**Conclusion:**

While BST-2 glycosylation was functionally irrelevant, formation of cysteine-linked dimers appeared to be important for inhibition of virus release. However lack of dimerization did not prevent surface expression or Vpu sensitivity of BST-2, suggesting Vpu sensitivity and inhibition of virus release are separable properties of BST-2.

## Background

Vpu is an 81 amino acid type 1 integral membrane protein [1,2] that has been shown to cause proteasomal degradation of CD4 [3,4] but also enhances the release of virions from infected cells [5-7]. These two biological activities of Vpu are mechanistically distinct and involve different structural domains in Vpu. In particular, two conserved phosphoserine residues in the cytoplasmic domain of Vpu (S52, S56) are crucial for CD4 degradation but have no or only a partial effect on virus release [8-11]. On the other hand, Vpu's transmembrane (TM) domain is critical for enhancement of particle release but it can be substituted by other membrane anchors without effect on CD4 degradation [12,13]

Previous data suggested that Vpu regulates the detachment of otherwise complete virions from the cell surface [5,14]. Subsequently, several mechanisms of Vpu mediated virus release have been proposed. First, a Vpu-associated ion channel activity was implicated in the regulation of virus release. Vpu has the ability to assemble into a monovalent cation-specific ion channel [15-19]. Randomization of Vpu's TM domain did not affect membrane association but inhibited Vpu's ion channel activity and, at the same time, impaired its ability to regulate virus release [12,17]. These observations established a correlation between Vpu ion channel activity and increased virus release activity. A second alternative model suggested that Vpu might interfere with the activity of Task-1, a cellular ion channel, through the formation of hetero-oligomeric complexes [20]. Overexpression of a dominant-negative fragment of Task-1 inhibited Task-1 ion channel activity and increased release of *vpu*-deficient particles thus creating a functional correlation between Task-1 ion channel activity and reduced HIV-1 particle release [20]. It is not known, however, if expression of Task-1 is tissue specific and it remains unclear, exactly how either Vpu or Task-1 ion channel activities might regulate detachment of particles from the cell surface.

A third model invokes the inactivation of a cellular inhibitor of virus release. This model is based on the observation that Vpu-dependent virus release is host cell-dependent [21]. Indeed, in addition to Task-1, several other host factors have been identified whose overexpression was associated with reduced virus release. These include the Vpu binding protein UBP [22], the recently identified host factors BST-2 (also referred to as tetherin, CD317, or HM1.24 [23,24]), and CAML [25]. Among those, BST-2 is of particular interest since its cell type-specific expression most closely matches that of Vpu-dependent cell types and silencing of BST-2 expression in HeLa cells by siRNA or shRNA rendered virus release from these cells Vpu-independent [23,24].

A functional Vpu-BST-2 interaction was first reported in a quantitative membrane proteomics study where Vpu expressed from an adenovirus vector was found to reduce cellular expression of BST-2 in HeLa cells [26]. Intriguingly, subsequent reports found that BST-2 expression varied in a cell type dependent manner; BST-2 mRNA was constitutively expressed in cell types such as HeLa, Jurkat, or CD4+ T cells but not 293T or HT1080 cells and thus corresponded to cell types known to depend on Vpu for efficient virus release [23,24]. Also, BST-2 expression was induced by interferon treatment in 293T and HT1080 cells [24] consistent with the previous observation that interferon treatment of various cell lines that did not normally require Vpu for efficient virus release became Vpu-dependent [27]. Additionally, ectopic expression of BST-2 in 293T or HT1080 cells rendered these cells Vpu dependent. This strongly suggested that BST-2 was indeed a host factor whose inhibitory effect on virus release was counteracted by Vpu [23,24].

**Figure 1:**
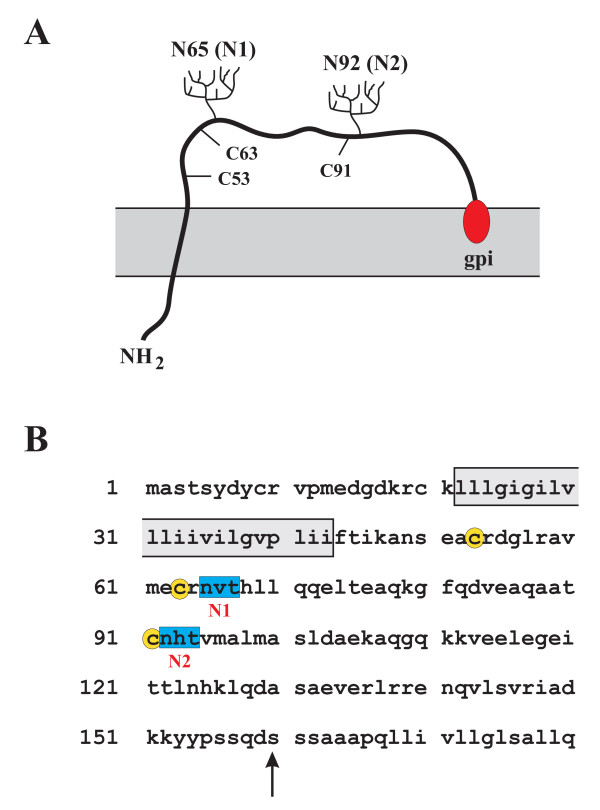
**Schematic of the BST-2 structure**. **(A) **BST-2 is a type 2 integral membrane protein. The N-terminus localizes to the cytoplasm. The BST-2 ectodomain contains three cysteine residues (C53, C63, C91) and two potential sites for N-linked glycosylation (N65, N92). The C-terminus of BST-2 is modified by the addition of a glycosyl-phosphatidylinositol (gpi) anchor. **(B) **Predicted amino acid sequence of human BST-2. The predicted transmembrane region is indicated by a box. Signals for N-linked glycosylation are marked in blue; cysteine residues in the BST-2 extracellular domain are highlighted in yellow. The arrow points to the predicted site of cleavage for the addition of the gpi anchor [31].

BST-2 was originally identified as a membrane protein in terminally differentiated human B cells of patients with multiple myeloma [28,29] BST-2 is a 30-36 kDa type II transmembrane protein, consisting of 180 amino acids [30]. The protein has both an N-terminal transmembrane domain and a C-terminal glycosyl-phosphatidylinositol (GPI) anchor (Fig. 1) [31]. BST-2 protein associates with lipid rafts at the cell surface and on internal membranes, presumably the TGN [31]. Also, BST-2 forms stable cysteine-linked dimers [29] and is modified by N-linked glycosylation [29,31]. However, the precise function of these BST-2 modifications remains unknown. N-linked glycosylation was dispensable for inhibition of Lassa and Marburg virus release, but the significance of BST-2 glycosylation has not been examined in relation to HIV-1 [32]. Recent data suggest that the BST-2 TM domain is critical for interference by Vpu [33-35]. consistent with previous observations of the importance of the Vpu TM domain for the regulation of virus release [12,13,36]. Furthermore, antagonism of BST-2 was reported to involve intracellular reduction of BST-2 levels by Vpu [37-40]. and was shown to encompass a β-TrCP-dependent endo-lysosomal pathway [38].

Here we analyzed the functional importance of various structural properties of BST-2. We show that both predicted N-linked glycosylation sites are utilized in the human protein. Interestingly, while endogenous BST-2 in HeLa cells and other cell types contained almost exclusively complex carbohydrate modifications, a large proportion of transiently expressed BST-2 was modified by high-mannose carbohydrates, a modification common to endoplasmic reticulum (ER) resident glycoproteins. Intriguingly, mutation of both glycosylation sites did not inhibit cell surface expression of BST-2 and neither abolished sensitivity to Vpu nor eliminated BST-2's inhibitory effect on HIV-1 particle release. Thus, carbohydrate modification of BST-2 does not appear to have any functional significance as far as HIV-1 virus release is concerned. In contrast, the formation of cysteine linked dimers of BST-2 appeared to be functionally important. We confirmed that BST-2 forms cysteine-linked dimers involving three cysteine residues in the extracellular domain. Mutation of individual cysteine residues or of any two of the three cysteine residues in combination failed to affect BST-2 dimerization and had no effect on BST-2's inhibition of virus release. In contrast, BST-2 mutated in all three cysteine residues was unable to inhibit HIV-1 virus release. Interestingly, this mutant was still expressed at the cell surface and remained sensitive to Vpu. The inability of the triple cysteine mutant to inhibit virus release was therefore not due to gross mislocalization or misfolding of the protein. Our results suggest that the formation of cysteine-linked dimers is a critical requirement for the inhibition of virus release by BST-2.

## Methods

### Plasmids

The full length infectious HIV-1 molecular clone pNL4-3 and the Vpu deletion mutant pNL4-3/Udel have been described [5,41] For transient expression of Vpu, the codon-optimized vector pcDNA-Vphu [42] was employed. Plasmid pcDNA-BST-2 is a vector for the expression of human BST-2 under the control of the cytomegalovirus immediate-early promoter. BST-2 was amplified by RT-PCR from HeLa mRNA using the primers 5' ATAAC TCGAG GTGGA ATTCA TGGCA TCTAC TTCGT ATGAC TATTGC and 3' AAGCT TGGTA CCTCA CTGCA GCAGA GCGCT GAGGC CCAGC AGCAC. The resulting PCR product was cleaved with *Xho*I and *Kpn*I and cloned into the *Xho*I/*Kpn*I sites of pcDNA3.1(-) (Invitrogen Corp., Carlsbad CA). Mutation of cysteine residues C53, C63, and C91 in human BST-2, either alone or in combination, to alanine was accomplished by PCR-based mutagenesis of pcDNA-BST-2 and resulted in pcDNA-BST-2 C53A, pcDNA-BST-2 C63A, pcDNA-BST-2 C91A, pcDNA-BST-2 C12 (C53,63A), pcDNA-BST-2 C13 (C53,91A), pcDNA-BST-2 C23 (C63,91A), and pcDNA-BST-2 C3A (C53,63,91A). Mutation of two potential N-linked glycosylation sites was similarly accomplished by PCR-based mutagenesis and resulted in the change of asparagine residues N65 and N92 to glutamine in human BST-2 either alone or in combination. PCR products were cloned into pcDNA-BST-2 to obtain pcDNA-BST-2 N1 (N65Q), pcDNA-BST-2 N2 (N92Q), and pcDNA-BST-2 N1/N2 (N65,92Q). The presence of the desired mutations and the absence of additional mutations were verified for each construct by sequence analysis.

### Antisera

Anti-BST-2 antiserum was elicited in rabbits by using a bacterially expressed MS2-BST-2 fusion protein composed of amino acids 1 to 91 of the MS2 replicase and amino acids 41 to 162 of BST-2 generating a polyclonal antibody against the extracellular portion of BST-2. Polyclonal anti-Vpu serum (rabbit), directed against the hydrophilic C-terminal cytoplasmic domain of Vpu expressed in *Escherichia coli *[43] was used for detection of Vpu. Serum from an HIV-positive patient was used to detect HIV-1-specific capsid (CA) and Pr55gag precursor proteins. Tubulin was identified using a monoclonal antibody to α-tubulin (Sigma-Aldrich, Inc., St. Louis MO).

### Tissue culture and transfections

HeLa and 293T cells were propagated in Dulbecco's modified Eagles medium (DMEM) containing 10% fetal bovine serum (FBS). For transfection, cells were grown in 25 cm^2 ^flasks to about 80% confluency. Cells were transfected using LipofectAMINE PLUS™ (Invitrogen Corp, Carlsbad CA) following the manufacturer's recommendations. A total of 5 μg of plasmid DNA per 25 cm^2 ^flask was used. Total amounts of transfected DNA was kept constant in all samples of any given experiment by adding empty vector DNA as appropriate. Cells were harvested 24 h post transfection.

### Immunoblotting

For immunoblot analysis of intracellular proteins, whole cell lysates were prepared as follows: Cells were washed once with PBS, suspended in PBS (400 μl/10^7 ^cells), and mixed with an equal volume of sample buffer (4% sodium dodecyl sulfate, 125 mM Tris-HCl, pH 6.8, 10% 2-mercaptoethanol, 10% glycerol, and 0.002% bromophenol blue). For analysis of cysteine mutants under non-reducing conditions, cells were suspended in PBS and mixed with an equal volume of sample buffer that did not contain 2-mercaptoethanol. Proteins were solubilized by boiling for 10 to 15 min at 95°C with occasional vortexing of the samples to shear cellular DNA. Residual insoluble material was removed by centrifugation (2 min, 15,000 rpm in an Eppendorf Minifuge). Cell lysates were subjected to SDS-PAGE; proteins were transferred to PVDF membranes and reacted with appropriate antibodies as described in the text. Membranes were then incubated with horseradish peroxidase-conjugated secondary antibodies (Amersham Biosciences, Piscataway NJ) and visualized by enhanced chemiluminescence (ECL, Amersham Biosciences).

### Metabolic labeling and immunoprecipitations

Cells were transfected as described in the text with constant amounts of proviral vectors and increasing amounts of BST-2. Twenty-four hours later, cells were washed with PBS, scraped and resuspended in 3 ml labeling media lacking methionine (Millipore Corp., Billerica MA). Cells were then incubated for 15 minutes at 37°C to deplete the endogenous methionine pool. After starvation cells were pelleted again, resuspended in 400 μl of labeling medium, and 150 μCi of [^35^S]-methionine was added to each sample. Cells were labeled for 90 minutes at 37°C. Then, cells were pelleted (20 sec, 10,000 × g). The virus-containing supernatant was removed and filtered through 0.45 μm cellulose acetate spin filters (Corning Costar Corp., Cambridge MA). Virions were lysed in 0.1% Triton-X100, 0.1% bovine serum albumin (BSA) in PBS. Cells were lysed in 200 μl of Triton lysis buffer (50 mM Tris pH 7.5, 150 mM NaCl, 0.5% Triton-X100) and incubated on ice for 5 minutes. After lysis, the cells were pelleted at 13,000 × g for 2 minutes to remove insoluble material. The supernatants and the virus lysates were incubated on a rotating wheel for 1 hr at 4°C with protein A-Sepharose coupled with an HIV-positive patient serum. Beads were washed twice with wash buffer (50 mM Tris pH 7.4, 300 mM NaCl, 0.1% Triton X-100). Bound proteins were eluted by heating in sample buffer for 10 min at 95°C, separated by SDS-PAGE, and visualized by fluorography.

### Concanavalin A (ConA) and datura stramonium lectin (DS lectin) binding assays

For glycoprotein analysis of BST-2, cell lysates were prepared as follows: Cells were washed once with PBS and lysed in 300 μl of lysis buffer (50 mM Tris pH 8.0, 100 mM NaCl, 5 mM ethylenediaminetetraacetic acid, 0.5% CHAPS) and 40 μl of DOC (2% deoxycholate in lysis buffer). The cell extracts were clarified at 13,000 × *g *for 2 min and the supernatant was incubated on a rotating wheel for 1-3 h at 4°C with ConA or DS lectin resin (Vector Laboratories, Burlingame CA) in 0.1% BSA-PBS. Complexes were washed three times with 50 mM Tris, 300 mM NaCl, and 0.1% Triton X100, pH 7.4. Bound proteins were eluted from beads by heating in sample buffer for 5 - 10 min at 95°C and analyzed by immunoblotting.

### Glycoprotein analysis

All digestions were performed directly on BST-2 bound to either ConA or DS lectin resin. Control reactions conducted in parallel did not contain enzyme. For endoglycosidase H (Endo H) and Peptide: N-Glycosidase F analysis (PNGase) beads were washed with denaturing buffer (New England BioLabs, Ipswich MA), then resuspended in denaturing buffer and boiled at 95°C for 10 min. The supplied reaction buffer was added along with 0.1% NP-40 according to the manufacturer's suggestion. An excess of enzyme, 2500 units of Endo H (New England BioLabs, Ipswich MA) or PNGase (New England BioLabs, Ipswich MA), was added and samples were digested at 37°C for 3 h. Bound proteins were eluted from beads by heating in an equal volume of sample buffer for 10 min at 95°C and analyzed by immunoblotting. For endo-β-galactosidase (Endo B) analysis, beads were first washed with 50 mM sodium acetate (pH 5.8), then resuspended in the same buffer supplemented with BSA to 0.1%. 5 mU of Endo B (Associates of Cape Cod, East Falmouth MA) was added and digested at 37°C for 16 h along with a control lacking the enzyme.

### FACS analysis

Cells were washed twice with ice-cold 20 mM EDTA-PBS, followed by 2 washes in ice-cold 1% BSA-PBS. Cells were treated for 10 min with 50 μg of mouse IgG (Millipore, Temecula CA) to block non-specific binding sites. Cells were incubated with primary antibody (α-BST-2) for 30 min at room temperature. Cells were then washed twice with ice-cold 1% BSA-PBS followed by addition of allophycocyanin (APC)-conjugated anti rabbit IgG secondary antibody (Jackson Immuno Research Lab Inc., West Grove PA) in 1% BSA-PBS. Incubation was for 30 min at room temperature in the dark. Cells were then washed twice with ice-cold 1% BSA-PBS and fixed with 1% paraformaldehyde in PBS. Finally, cells were analyzed on a FACS Calibur (BD Biosciences Immunocytometry Systems, Mountain View CA). Data analysis was performed using Flow Jo (Tree Star, San Carlos CA). For gating of transfected cells, pEGFP-N1 (Clontech, Mountain View CA) was cotransfected.

## Results

### Endogenous and exogenously expressed BST-2 have distinct glycosylation profiles

**Figure 2:**
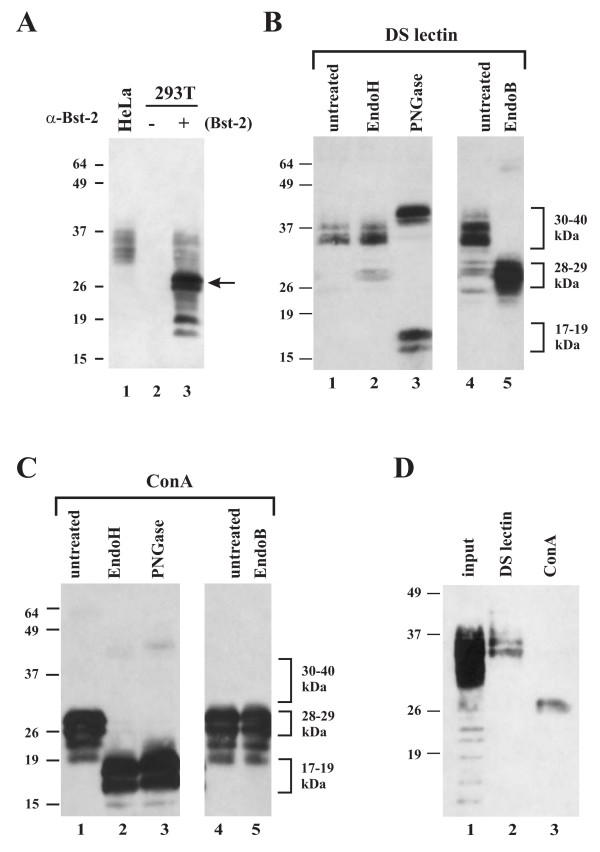
**Comparison of endogenous BST-2 in HeLa cells to BST-2 expressed in transiently transfected 293T cells**. **(A) **293T cells were transfected with wt BST-2 (lane 3). A mock transfected culture from HeLa (lane 1) and 293T cells (lane 2) was analyzed in parallel. Whole cell lysates were processed for immunoblotting as described in Methods. The arrow identifies a BST-2 species in transfected 293T cells not seen in HeLa cells. **(B & C) **Endoglycosidase analysis of transiently expressed BST-2. 293T cells were transfected with pcDNA-BST-2. BST-2 was enriched by adsorption to either datura stramonium lectin resin (DS lectin) **(B) **or Concanavalin A resin (ConA) **(C) **as described in Methods. DS lectin or ConA bound proteins were either left untreated (lanes 1 & 4) or treated with endoglycosidase H (EndoH) (lanes 2), Peptide: N-Glycosidase F (PNGase) (lanes 3), or endo-β-galactosidase (EndoB) (lanes 5) as described in Methods. Proteins were visualized by immunoblot analysis using a BST-2 specific antibody. **(D) **HeLa extracts were adsorbed to DS lectin (lane 2) and ConA resin (lane 3) as described for panels B & C. Total input lysate is shown in lane 1. A high mannose form of endogenous BST-2 was enriched on the ConA resin.

The biochemical characterization of BST-2 necessitates exogenous expression of the protein. For that purpose, the BST-2 gene was PCR-amplified from HeLa cell mRNA and cloned under the control of the cytomegalovirus immediate-early promoter as described in Methods. Ectopic expression of BST-2 from pcDNA-BST-2 was analyzed by immunoblot analysis of transiently transfected 293T cells using a BST-2-specific antibody (Fig. 2A, lane 3). HeLa cells expressing endogenous BST-2 (Fig. 2A, lane 1) and mock-transfected 293T cells (Fig. 2A, lane 2) were analyzed in parallel. Endogenous BST-2 in HeLa cells appeared as a smear of multiple bands with an apparent *M*r of 30-40 kDa, presumably due to N-linked glycosylation. Consistent with a previous report [24], untransfected 293T cells did not reveal BST-2 expression (Fig. 2A, lane 2). Of note, the bulk of transiently expressed BST-2 in 293T cells exhibited faster mobility in the gel than the endogenous protein and had an apparent *M*r of 28-29 kDa (Fig. 2A, lane 3, arrow). Only a minor fraction of the exogenously expressed BST-2 protein exhibited an electrophoretic mobility comparable to that of the endogenous protein in HeLa cells. The appearance of faster migrating forms of exogenously expressed BST-2 is not cell type-specific and was observed in transiently transfected HeLa cells as well (data not shown). Titrating transfected BST-2 DNA to the limit of detection in 293T cells did not prevent the appearance of the faster migrating forms of BST-2 (data not shown). Thus, the appearance of faster migrating forms of BST-2 in transiently transfected 293T cells are presumably a result of transient expression rather than protein overexpression per se.

### Exogenously expressed BST-2 contains high-mannose as well as complex carbohydrate modifications

It has been previously reported that rodent BST-2 is glycosylated, however the type of glycosylation has not been examined [29,31] Smeared protein patterns similar to BST-2 were previously observed for proteins with complex carbohydrate modifications [44] and it was likely that the 30-40 kDa and 28-29 kDa forms of BST-2 detected in transfected 293T cells above represented various carbohydrate modifications. We therefore performed an endoglycosidase analysis of transiently expressed BST-2. Enzymatic reactions were performed on BST-2 that was previously enriched by adsorption to either Concanavalin A (ConA) or datura stramonium lectin (DS lectin) resin. ConA recognizes α-linked high-mannose oligosaccharides, which are intermediates in N-linked glycosylation and are typically found on proteins that have not yet exited the endoplasmic reticulum (ER); DS lectin on the other hand binds β1-4 linked N-acetylglucosamine or N-acetyllactosamine repeats, which are characteristic of fully processed oligosaccharides and produce the smear pattern on protein gels noted above [44]. The latter modifications are typically found on glycoproteins that have exited the ER (for review see [45]). Peptide:N-Glycosidase F (PNGase) cleaves glycoproteins between the innermost GlcNAc and asparagine residues of all oligosaccharides from N-linked glycoproteins [46]. Therefore all N-linked oligosaccharides will be sensitive to PNGase treatment. Endo-β-N-acetylglucosaminidase H (EndoH), on the other hand, is more selective than PNGase and cleaves the chitobiose core of high-mannose from N-linked glycoproteins [46]. Because of that, ER associated proteins are generally sensitive to EndoH treatment. Proteins exiting the ER to the Golgi typically undergo additional sugar modifications and, as a result, become EndoH resistant. A third type of endoglycosidase, endo-β-galactosidase (EndoB), cleaves glycoproteins after β-galactosidic linkages [47] typically observed on glycoproteins that have exited the ER. Accordingly, glycoproteins residing in the ER are generally EndoB resistant.

As expected, PNGase treatment removed all oligosaccharides (Fig. 2B &2C, lane 3) resulting in deglycosylated proteins with a *M*r of 17-19 kDa and appeared as a doublet. The reason why deglycosylated BST-2 runs as a doublet is not clear but could be due to other modifications such as phosphorylation or the presence and absence of the GPI anchor. PNGase-treated BST-2 samples adsorbed to DS lectin columns revealed an additional protein doublet of 38-40 kDa (Fig. 2B, lane 3). The precise nature of this protein species is unclear but its mobility in the gel is consistent with that predicted for a dimer form of BST-2. BST-2 enriched by DS lectin columns was largely resistant to EndoH treatment (Fig. 2B, compare lanes 1 & 2). EndoH resistance indicated that the 30-40 kDa population of BST-2 contained complex sugar modifications and therefore has likely exited the ER. This was confirmed by their sensitivity to EndoB treatment (Fig. 2B, lane 5). In contrast, the 28-29 kDa population of BST-2 enriched by ConA was highly sensitive to EndoH (Fig. 2C, lane 2) but was resistant to EndoB treatment (Fig. 2C, lane 5). These results suggest that the 28-29 kDa protein population observed in transfected 293T cells represents a high-mannose form of BST-2. Therefore, exogenously expressed BST-2 consists of two populations: a 30-40 kDa form containing complex sugar modifications (referred to as "post-ER form" for the remainder of the text) and a predominant 28-29 kDa population containing high-mannose oligosaccharide modifications (referred to as "high-mannose form" in the following). HeLa cells expressing endogenous BST-2 were not entirely devoid of the high-mannose form of BST-2. However, the high-mannose form of endogenous BST-2 was detectable in HeLa cells only on over-exposed gels (compare lanes 1 in Figs. 2A &2D) but could be enriched by adsorption of cell lysates to ConA lectin (Fig. 2D, lane 3).

### BST-2 is glycosylated at asparagine residues 65 and 92

**Figure 3:**
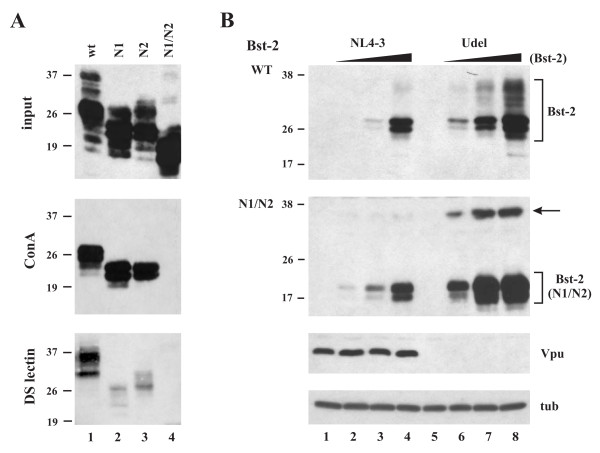
**Immunoblot analysis of BST-2 glycosylation mutants**. **(A) **293T cells were transfected with wt BST-2, single glycosylation site mutants N1 & N2, or the double glycosylation site mutant N1/N2. BST-2 specific proteins were identified by immunoblotting using a BST-2-specific polyclonal antibody (top panel). Aliquots of the same samples were adsorbed to either ConA (middle panel) or DS lectin (lower panel) as described in Methods. Eluates were analyzed by immunoblotting using a BST-2-specific polyclonal antibody. **(B) **293T cells were transfected with 5 μg each of NL4-3 wt (lanes 1-4) or NL4-3/Udel (lanes 5-8) either in the absence of BST-2 (lanes 1 & 5) or in the presence of 0.01 μg (lanes 2 & 6), 0.03 μg (lanes 3 & 7), or 0.1 μg (lanes 4 & 8) BST-2 DNA. Cells were harvested 20 h post transfection. A fraction of the cells was used for immunoblot analysis; the other part was used for metabolic labelling (Fig. 4). Whole cell lysates were prepared and used for immunoblot analysis using a BST-2-specific polyclonal antibody (top two panels). The blots were then sequentially reprobed with antibodies to Vpu (third panel) or tubulin (lower panel). Representative samples shown in the lower panels were from the N1/N2 blot. Proteins are identified on the right. The arrow points to a form of BST-2 N1/N2 whose migration in the gel is consistent with a dimer.

The predicted amino acid sequence for BST-2 contains two potential N-linked glycosylation sites at positions 65 and 92 of the 180 residue protein [30]. Previous studies reporting on rodent BST-2 glycosylation did not clearly address whether one or both of the two potential N-linked glycosylation sites in the BST-2 ectodomain were modified [31]. To address this issue we mutated asparagine residues 65 and 92 of human BST-2 to glutamine. Mutations were introduced either individually into BST-2 to result in mutants N1 (N65Q) and N2 (N92Q), respectively, or in combination to result in mutant N1/N2 (N65,92Q). Mutants were expressed in 293T cells and analyzed by immunoblotting using a BST-2-specific antibody. As shown in figure 3A, mutation of individual glycosylation sites reduced the apparent *M*r of the N1 as well as the N2 mutant relative to the wild type (wt) protein (Fig. 3A, top panel; lanes 1-3). Mutation of both potential glycosylation sites in the double mutant (N1/N2) further reduced the apparent *M*r of the predominant 28-29 kDa species to about 17-19 kDa (Fig. 3A, top panel; lane 4) closely matching the predicted *M*r of 19.7 kDa for non-glycosylated BST-2. These results indicate that both predicted glycosylation sites at BST-2 residues N65 and N92 are modified.

The use of both glycosylation sites was further verified by a pull-down experiment using ConA resin (Fig. 3A, middle panel) and DS lectin resin (lower panel). As can be seen, wt BST-2 and the N1 and N2 single glycosylation mutants interacted with ConA and DS lectin. As predicted, the N1/N2 double mutant had no affinity to ConA or DS lectin due to the lack of oligosaccharide modification of this BST-2 mutant.

### Glycosylation of BST-2 is not required for inhibition of virus release and sensitivity to Vpu

To assess the importance of BST-2 glycosylation for the inhibition of virus release and for sensitivity to Vpu (i.e. reduction of BST-2 steady state levels) we performed a set of experiments, in which the release of metabolically labeled viral Gag proteins was determined in the presence of increasing amounts of wt BST-2 or the N1/N2 BST-2 double glycosylation mutant. For that purpose wt NL4-3 or NL4-3/Udel were transfected into 293T cells either alone or in combination with wt BST-2 or BST-2 N1/N2 at virus:BST-2 ratios of 500:1 to 50:1. Prior to labelling, a portion of the transfected cells was removed to allow simultaneous analysis of intracellular BST-2 expression by immunoblotting. As shown in figure 3B transfection of increasing amounts of BST-2 DNA resulted in the dose-dependent increase in BST-2 expression (lanes 2-4 & 6-8). BST-2 wt and N1/N2 were expressed at comparable levels but exhibited different mobilities in the gel. Like the PNGase-treated samples in figure 2B, a sub-population of non-glycosylated N1/N2 protein exhibited mobility in the gel predicted for a BST-2 dimer (Fig. 2B, arrow). As reported previously [37], Vpu has the ability to reduce the expression of BST-2 [37-40]. Expression of Vpu in the samples was verified by immunoblotting (Fig. 3B, Vpu) and equal sample loading was verified by reprobing the blot with a tubulin-specific antibody (Fig. 3B, tub). Of note, levels of wt BST-2 as well as the N1/N2 mutant were lower in samples cotransfected with wt NL4-3 than in the corresponding NL4-3/Udel samples (compare lanes 2-4 & 6-8). This suggests that BST-2 N1/N2 expression, like wt BST-2, is sensitive to Vpu. Both the high mannose and post-ER form of BST-2 were sensitive to Vpu.

**Figure 4:**
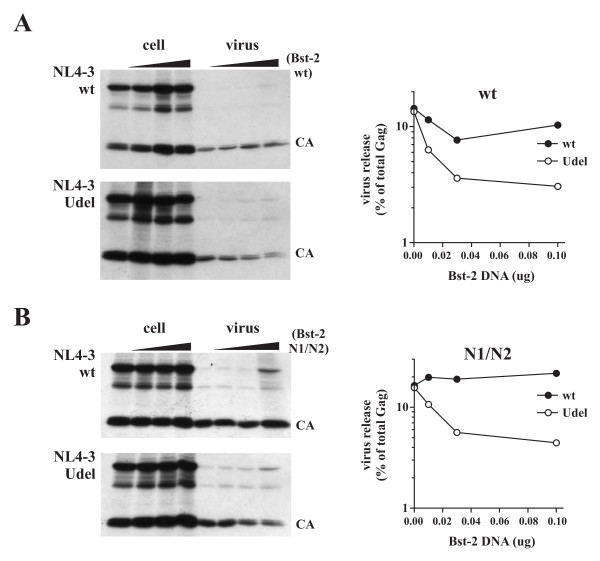
**Glycosylation of BST-2 is not required for inhibition of virus release and for sensitivity to Vpu**. **(A) **Analysis of wt BST-2. **(B) **Analysis of BST-2 N1/N2. **(A & B) **Cells were metabolically labeled for 90 min with [35S]-methionine as described in Methods and cell lysates and cell-free supernatants were subjected to immunoprecipitation by an HIV-positive patient serum. Immunoprecipitates were subjected to SDS-PAGE and proteins were visualized by fluorography (left panels). Virus release was quantified by phosphoimage analysis using a Fujifilm FLA7000. Virus release was calculated independently for each sample by determining the percentage of cell-free CA protein relative to the total intra- and extra-cellular Gag protein. Solid circles represent wt NL4-3. Open circles represent NL4-3/Udel. Data are presented as a function of BST-2 concentration on a semi-log plot.

To assess the importance of glycosylation on BST-2's ability to inhibit virus release, transfected cells were labeled for 90 min with [^35^S]-methionine and cell lysates and cell-free virus-containing supernatants were immunoprecipitated with an HIV-positive patient serum as described in Methods. Samples were separated by SDS-PAGE and immunoprecipitated proteins were visualized by fluorography (Fig. 4A &4B, left panels). Virus release was quantified by phosphoimage analysis (Fig. 4A &4B, right panels). The amount of viral protein in the cell-free supernatant was calculated as percentage of the total intra- and extra-cellular protein and was plotted as a function of BST-2 concentration on a semi-log plot. In the absence of BST-2 between 10-20% of total Gag protein was released within the 90 minute window of this experiment. As expected, wt BST-2 significantly reduced the release of *vpu*-deficient HIV-1 virions into the culture supernatants in a dose-dependent manner while it had no significant effect on the release of wt virus (Fig. 4A). Interestingly, the N1/N2 BST-2 glycosylation mutant inhibited release of Vpu-deficient virus to a similar extent as wt BST-2 and did not inhibit release of wt NL4-3 (Fig. 4B). We therefore conclude that glycosylation of BST-2 is not critical for the inhibition of virus release and does not affect sensitivity of BST-2 to Vpu.

### Any one of three cysteines in the BST-2 ectodomain can mediate BST-2 dimerization

**Figure 5:**
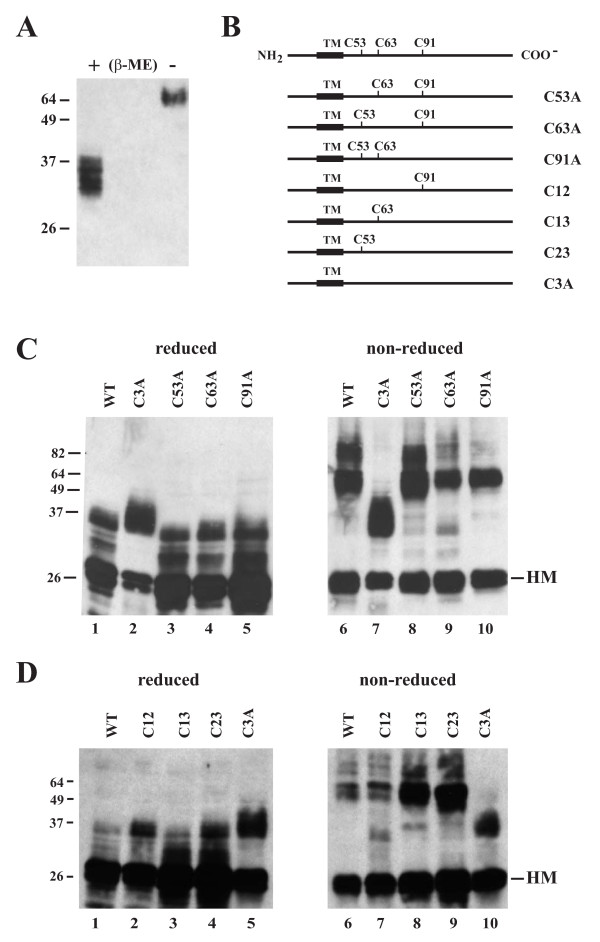
**BST-2 forms cysteine-linked dimers**. **(A) **HeLa cells were lysed in reducing (+β-ME) or non-reducing (-β-ME) sample buffer as described in Methods. Lysates were separated by SDS-PAGE and subjected to immunoblot analysis using a BST-2-specific polyclonal antibody. **(B) **Schematic representation of mutants analyzed in this experiment. In all cases, cysteine residues were mutated to alanine by site-directed mutagenesis. Remaining cysteine residues are shown for each mutant. **(C) **293T cells were transfected with wt BST-2 (lanes 1 & 6), the triple cysteine mutant C3A (lanes 2 & 7), or individual cysteine mutants (lanes 3-5 & 8-10). Cells were harvested 24 h post transfection, washed in PBS, and split into two equal samples. One set of samples was mixed with an equal volume of reducing sample buffer (lanes 1-5); the second set was mixed with sample buffer lacking β-ME (lanes 6-10). Cell lysates were separated by SDS-PAGE and subjected to immunoblotting using a BST-2 specific antibody. **(D) **293T cells were transfected with wt BST-2 (lanes 1 & 6), the triple cysteine mutant C3A (lanes 5 & 10), or double-cysteine mutants (lanes 2-4 & 7-9). Samples were analyzed under reducing and non-reducing conditions as in panel C. (HM) marks the position of the high-mannose forms of BST-2 in the gels.

In addition to glycosylation, other predicted modifications in BST-2 arise from the ability to homo-dimerize [29]. We verified this property of BST-2 by analyzing endogenous BST-2 from HeLa cells under reducing and non-reducing conditions. HeLa extracts were prepared as described in the Methods section and separated by SDS-PAGE. To prevent inadvertent reduction of the non-reduced sample by the mercapto-ethanol present in the reduced sample, samples were separated by two empty lanes in the gel. As can be seen in figure 5A, BST-2, when prepared under non-reducing conditions (- β-ME), quantitatively shifted into a slower migrating form with an *M*r of ~65-70 kDa consistent with the predicted position of a BST-2 dimer.

BST-2 contains three cysteine residues in its extracellular domain that could be involved in the formation of cysteine-linked dimers (Fig. 1). We mutated the cysteines at positions 53, 63, and 91 individually or in combinations of two to alanine to obtain mutants C53A, C63A, C91A, C12, C13, and C23, respectively. In addition, we created a mutant, C3A, in which all three of these cysteines were changed to alanine. The mutants are schematically depicted in figure 5B. The ability of these mutants to form cysteine-linked homo-dimers was tested in transiently transfected 293T cells. All cysteine mutants produced a dominant 28-29 kDa high-mannose form of BST-2 (Fig. 5C/D, HM) in addition to the 30-40 kDa post-ER form carrying complex sugar modifications. The combined mutation of all three extracellular cysteines in C3A affected the mobility of the post-ER form of BST-2 resulting in a more compressed, less smeared pattern (Fig. 5C lane 2; 5D, lane 5). The reason for this is unclear but could be indicative of altered oligosaccharide processing of monomeric BST-2. Protein analysis under non-reducing conditions demonstrated that mutation of individual cysteines had no impact on the ability of BST-2 to homo-dimerize (Fig. 5C, lanes 8-10). Similarly, mutation of two of the three cysteine residues in any combination did not affect BST-2 dimerization (Fig. 5D, lanes 7-9). Equal amounts of DNA were transfected in all samples. Nevertheless, relative protein levels in the samples varied, perhaps due to differences in protein stability or antibody recognition. As predicted, mutation of all three cysteines abolished BST-2 dimerization (Fig. 5C, lane 7; 5D, lane 10). These results suggest that any one of three cysteines in the BST-2 extracellular domain can mediate BST-2 dimerization. Finally, it is interesting that the relative prevalence of the high-mannose (HM) form of BST-2 did not change under non-reducing conditions, while the post-ER form of BST-2 is no longer present at the same size under non-reducing conditions suggesting that only the post-ER form of BST-2 can efficiently homo-dimerize while the high mannose form remains largely, if not exclusively, in monomeric form.

### BST-2 dimerization is important for inhibition of virus release

**Figure 6:**
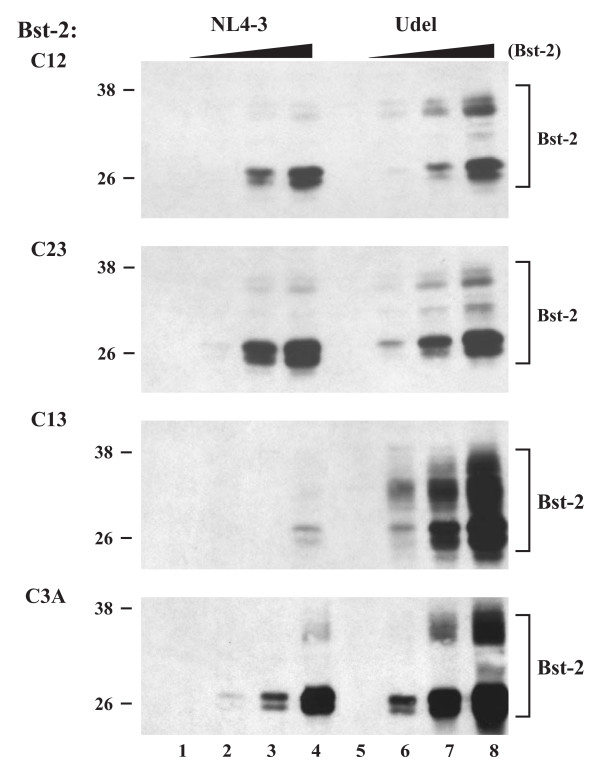
**Dimerization of BST-2 is not a prerequisite for sensitivity to Vpu**. 293T cells were transfected with 5 μg of wt pNL4-3 (lanes 1-4) or pNL4-3/Udel (lanes 5-8) in the absence of BST-2 (lanes 1 & 5) or together with 0.01 (lanes 2 & 6), 0.03 (lanes 3 & 7), and 0.1 μg (lanes 4 & 8) of vectors encoding cysteine mutants of BST-2 containing only one remaining cysteine (C12, C23, C13) or no cysteine at all (C3A). Twenty-four hours later, cell lysates were prepared from a fraction of the cells as described in the Methods and subjected to immunoblot analysis using a BST-2-specific antibody.

**Figure 7:**
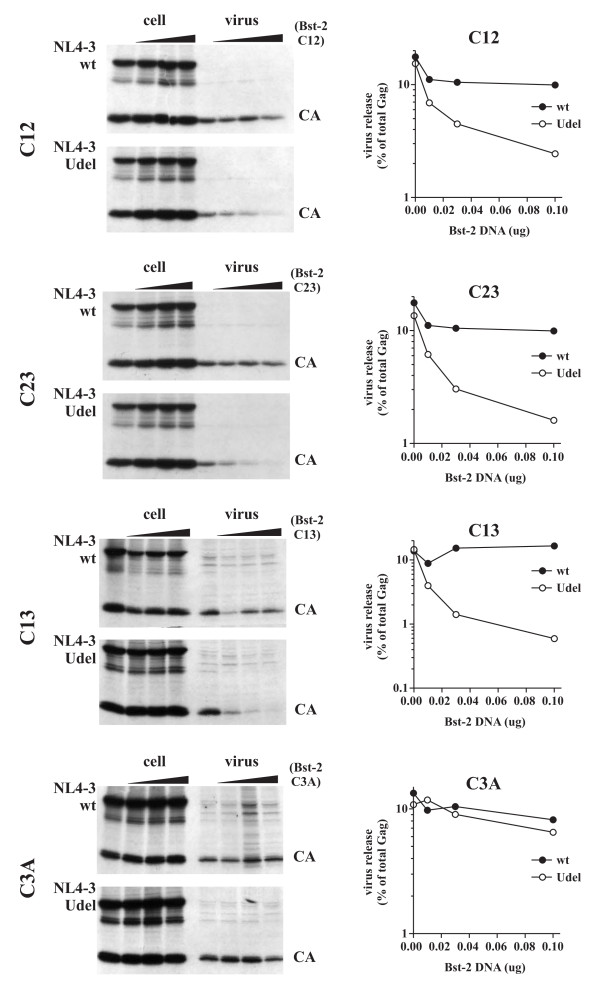
**Dimerization of BST-2 is important for inhibition of virus release**. Transfected 293T cells from figure 6 were metabolically labeled for 90 minutes and analyzed as described for figure 4. Proteins were identified by fluorography (left panels). Quantification of virus release was performed as described for figure 4 and is shown on the right.

The importance of cysteine-linked dimerization of BST-2 for inhibition of virus release was assessed by metabolic labeling as described for the glycosylation mutants (Fig. 4) by coexpressing increasing amounts of BST-2 mutants with constant amounts of wt NL4-3 or NL4-3/Udel. At the same time, effects of Vpu on steady-state expression of BST-2 cysteine mutants were assessed by immunoblotting of cell lysates. Because double-cysteine mutants retained the ability to form cysteine linked dimers, individual cysteine mutants were ignored here and only double- and triple-cysteine mutants were tested for the ability of Vpu to decrease Bst-2 expression or to inhibit virus release. As observed for the glycosylation mutants, intracellular expression of the double- and triple-cysteine mutants was sensitive to Vpu to varying degrees (Fig. 6). In particular the C13 and C3A mutants were highly sensitive to Vpu similar to wt BST-2 while expression of the C12 and C23 mutants appeared to be less affected by the presence of Vpu. The reason for the differential sensitivity of these mutants to Vpu is unclear. Metabolic labelling followed by immunoprecipitation with an HIV-positive patient serum revealed that all of the double-cysteine mutants inhibited the release of Vpu-defective virus in a dose-dependent manner (Fig. 7; top three panels). In contrast, mutation of all three cysteines in the BST-2 ectodomain abolished its ability to inhibit virus release (Fig. 7, bottom panel). Inhibition of virus release by the cysteine mutants of BST-2 was specific since none of the mutants inhibited the release of wt virus. We therefore conclude that cysteine-linked homo-dimerization of BST-2 is important for the inhibition of HIV-1 virus release. These results also indicate that BST-2 dimerization is not dependent on one specific cysteine residue but can be mediated by any one of the three cysteine residues present in the protein's ectodomain. Because of the presence of three dimerization-competent cysteine residues, it is conceivable that BST-2 forms cysteine-linked homo-oligomeric structures. Studies are ongoing to investigate this possibility.

### Lack of cysteine-linked dimerization and carbohydrate modification of BST-2 does not preclude cell surface expression and surface downregulation by Vpu

**Figure 8:**
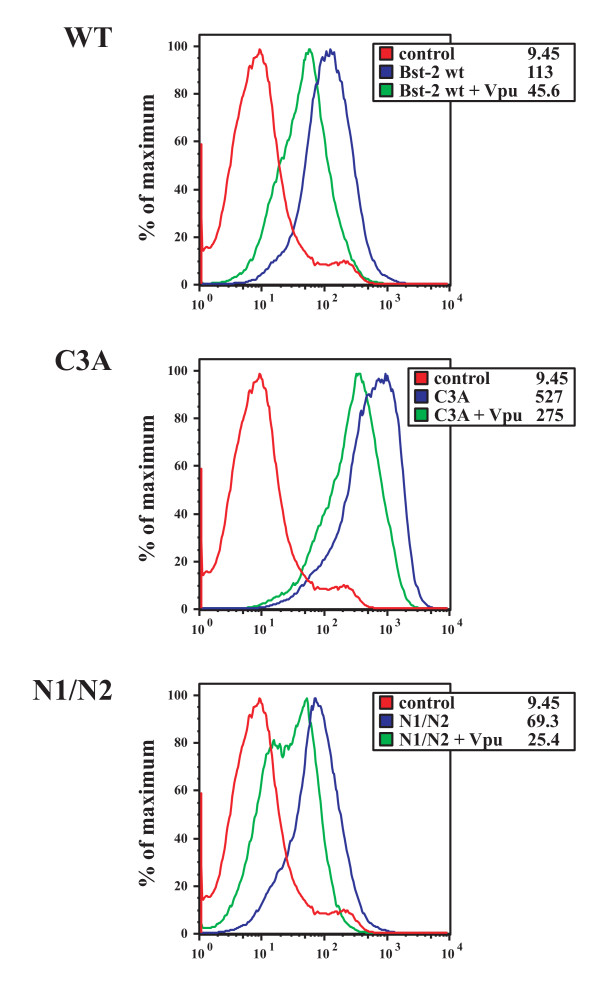
**Monomeric BST-2 and non-glycosylated BST-2 are expressed at the cell surface and are sensitive to Vpu-induced down modulation**. 293T cells were transfected with 0.1 μg each of wt BST-2, BST-2 C3A, or BST-2 N1/N2 together with 1 μg each of pEGFP-N1 in the presence or absence of 1 μg pcDNA-Vphu. All samples were adjusted to 5 μg total DNA with empty vector DNA. Cells were harvested 24 h after transfection and stained with an antibody to BST-2. As a control, 293T cells were transfected with pEGFP-N1 and empty vector in the absence of BST-2 and stained with BST-2 antibody (Ctrl). Samples were subjected to FACS analysis as described in Methods and gated for GFP-positive cells. The red line represents the control of GFP^+^/BST-2^- ^cells. Blue lines represent BST-2 staining in the absence of Vpu and green lines indicate BST-2 staining in the presence of Vpu. Numbers in the boxed legends represent mean fluorescence intensities for each sample.

We and others have previously reported that Vpu does have the ability to down-regulate BST-2 from the cell surface [23,26,33,37,37-40]. The inability of the BST-2 C3A mutant to inhibit virus release could be caused by cellular mislocalization resulting in lack of surface expression. To test this possibility we analyzed cell-surface expression and Vpu-dependent cell-surface down modulation in transiently transfected 293T cells. Vpu was expressed from a codon-optimized vector [42] together with wt BST-2 or the C3A or N1/N2 mutants (Fig. 8). A GFP expression vector was cotransfected to allow gating of transfected cells. This experimental setup was previously shown to be a reliable assay system for studying Vpu-induced BST-2 cell surface down modulation [23,37] FACS analysis revealed that both the cysteine-deficient BST-2 (C3A) and the non-glycosylated BST-2 (N1/N2) were expressed at the surface of transfected 293T cells (Fig. 8; blue lines) and were down-modulated by Vpu (Fig. 8; green lines). The mean fluorescence intensity of cell-surface C3A was higher in this experiment than that of wt BST-2 (Fig. 8, numbers in insets represent mean fluorescence intensity), perhaps due to increased protein stability or stronger affinity to the BST-2 antibody (or both). This was not further investigated as it does not affect the overall conclusion from this experiment, which is that neither N-linked glycosylation nor BST-2 dimerization are required for cell-surface expression and down-modulation by Vpu. These results further support the notion that inhibition of virus release and sensitivity to Vpu are independent properties of BST-2.

## Discussion

The identification of BST-2/tetherin as a Vpu-sensitive host factor has spurred new interest in the role of Vpu during HIV-1 particle release. Neil et al proposed a tethering mechanism where BST-2 acts in the manner of a bifunctional crosslinker to physically link otherwise fully detached virions to the cell surface or to each other [24]. Such a mechanism is very attractive but would be difficult to explain without virus-association of BST-2. Experimental proof for the tethering model remains to be established. Our own analyses have thus far failed to identify mature forms of BST-2 in virion preparations recovered from the surface of transfected HeLa cells by physical shearing [37]. It is of course possible that virus-associated amounts of mature BST-2 are simply below the limit of detection even though we were able to detect low levels of non-specifically secreted mature BST-2 [37]. In this regard it is interesting to note that high-mannose forms of BST-2, which are heavily overexpressed relative to mature BST-2 in transiently transfected 293T cells (e.g. figure 2A), were indeed observed in cell-free virion preparations (data not shown). However, the significance of this observation remains unclear since these BST-2 forms are not readily seen under physiological conditions. High mannose modifications are typical of ER-resident glycoproteins. However, there are a few examples, in which high mannose-modified glycoproteins were identified at the cell surface [48,49] Therefore, more work will be required to fully understand how BST-2 traffics to the cell surface and how it inhibits the release of HIV-1 virions. It also remains to be shown how Vpu enables the virus to bypass the inhibitory effect of BST-2. We confirmed that Vpu can cause surface downmodulation and reduction of intracellular levels of BST-2. While it is not entirely clear whether these effects of Vpu are prerequisites to efficient virus release [37], they provide useful analytical tools and were used here to test the sensitivity of BST-2 mutants to Vpu *in vitro*.

To get a better understanding of BST-2 and its role in HIV-1 virus release, we performed a biochemical characterization of BST-2 and studied how the biochemical properties relate to BST-2 function. BST-2 is a transmembrane glycoprotein that forms cysteine-linked homo-dimers and is expressed at the cell surface. All of our experiments were done with authentic untagged protein; yet, transiently expressed BST-2 exhibited dramatically different mobility on SDS PAGE than endogenous protein expressed in HeLa cells. We found that a large portion of transiently expressed BST-2 remained in a high-mannose form typical of ER associated proteins. The high-mannose form of BST-2 is not typically seen for endogenously expressed BST-2 in HeLa cells but can be induced by treatment with brefeldin A (BFA), a drug known to block export of membrane proteins from the ER (data not shown) and can be enriched by adsorbing lysates of untreated HeLa cells to ConA lectin (Fig. 2D). Reducing amounts of transfected DNA did not prevent accumulation of the high-mannose form in transient expression studies (e.g. Figs. 3B &6) indicating that it represents a relatively dose-independent phenomenon. Also, the effect was cell type-independent and seen in 293T and transiently transfected HeLa cells. Importantly, the relative abundance of high mannose BST-2 in transiently transfected 293T cells relative to endogenous BST-2 in HeLa cells did not abolish BST-2 function, as transiently expressed protein was capable of specifically and efficiently inhibiting virus release in the absence but not in the presence of Vpu. It is currently unclear if the accumulation of the high-mannose form of transiently expressed BST-2 is caused by a bottleneck effect delaying or preventing exit of BST-2 from the ER or whether BST-2 can exit the ER and bypass the complex sugar modification machinery of the Golgi altogether. On the other hand, we were able to detect high-mannose modified BST-2 in cell free virus preparations (data not shown), which could suggest that some of the protein is capable of trafficking to the cell surface without acquiring complex sugar modifications in the ER. While there is precedent in the literature for such a scenario [48,49], it is equally possible that extracellular BST-2 in such experiments was inadvertently released from a small number of cells that might have been destroyed during sample preparation. While we have referred to the different forms of BST-2 as "high-mannose" and "post-ER", we cannot unambiguously conclude that the high-mannose form of BST-2 represents an ER-resident form since we do not have reagents to discriminate the various forms of BST-2 by FACS or IFA. However, our observation of the functional importance of cysteine-linked dimers (discussed below), combined with the observation that the high-mannose form of BST-2 does not appear to dimerize, could imply that the high-mannose form of BST-2 is functionally inert. The biological role of BST-2 carrying high-mannose carbohydrate modifications will require additional investigation; however, we can already conclude that glycosylation per se is neither required for protein exit from the ER nor for cell surface presentation. This conclusion is based on our observation that the glycosylation-deficient N1/N2 mutant was not only expressed at the cell surface but inhibited the release of Vpu-deficient HIV-1 virions with similar efficiency as wt BST-2 and was sensitive to downregulation by Vpu (Figs. 4 &8).

Interestingly, while glycosylation of BST-2 appears to be functionally insignificant, formation of cysteine-linked dimers was important for BST-2's inhibitory effect on virus release. BST-2 encodes three cysteines in its ectodomain. Our results suggest that formation of cysteine-linked dimers is not mediated by any one specific cysteine residue in the ectodomain since mutation of each cysteine individually or in combination of two did not affect protein dimerization. The fact that none of these mutations affected BST-2 function supports the notion that the loss of activity seen for the triple cysteine mutant is due to the lack of protein dimerization rather than caused by a change in the amino acid sequence. The fact that the BST-2 triple cysteine mutant C3A was expressed at the cell surface and that cell surface expression was sensitive to Vpu (Fig. 8) further suggests that the lack of dimerization does not critically impair intracellular trafficking of the mutant protein. BST-2 dimerization could favor a tethering model since protein dimerization might have a stabilizing effect and strengthen the link between viral and cellular membranes. Studies on Ebola virus release found that treatment of cells with up to 500 mM DTT did not reverse the inhibitory effect of BST-2 [50]. While this could suggest that BST-2 dimers are not functionally important, it is equally possible that these conditions were not sufficient to reduce the BST-2 dimers. In some of our experiments, BST-2 dimers were seen even after boiling of the samples in reducing buffer (e.g. Fig. 2B, lane 3; Fig. 3B). Thus, BST-2 dimers appear to be quite stable and the effects of reducing agents on the dimer structure of BST-2 and on the shedding of *vpu*-deficient particles from HeLa cells will be interesting to investigate in future studies.

Finally, assuming BST-2 inhibits virus release from the cell surface we would predict that BFA treatment, which can trap BST-2 in the ER, will render virus release from HeLa cells Vpu-independent. However, in a previous study we found that virus release in BFA-treated HeLa cells was reduced to the level of untreated Vpu-negative cells irrespective of the presence or absence of Vpu [51]. Thus, BFA treatment did not render virus release from HeLa cells Vpu-independent but, quite to the contrary, Vpu insensitive. It is not clear at this time whether BFA treatment prevents Vpu from reaching its proper intracellular location required for targeting BST-2 or whether a 4 hour BFA-treatment cannot sufficiently reduce surface expression of BST-2. Experiments are ongoing to solve this conundrum. Our experiments do not address the question where in the cell BST-2 functions to inhibit virus release, however the high-mannose form of BST-2 appeared to be less sensitive to Vpu than the post-ER form (Figures 3 &6). Dube et al recently reported that regulation of virus release correlates with localization of Vpu in the trans-Golgi network [52]. Our own previous experiments have identified Vpu at the cell surface of transfected HeLa cells although cell surface levels of Vpu are likely to be low [53]. Thus, Vpu could act at the cell surface to displace BST-2 from the site of virus budding or it could act from within the cell by affecting BST-2 trafficking [38,52] Deciphering the mechanism of Vpu function will be a major focus of our future work in this area.

## Conclusion

Our study provides first insights into the functional importance of cysteine-linked dimers of BST-2 while at the same time demonstrating the relative unimportance of N-linked glycosylation for the inhibition of virus release. Interestingly, the loss of activity of a BST-2 mutant unable to form cysteine-linked dimers was not caused by lack of cell surface expression. Also, the dimerization mutant remained sensitive to Vpu, thus ruling out gross mislocalization and misfolding of the protein. We therefore conclude that formation of cysteine-linked BST-2 dimers is a functional requirement for inhibition of virus release. Finally, we observed that transient expression of BST-2 leads to the production of a dominant high mannose form of BST-2 that is present but underrepresented in the pool of endogenous protein. Although high mannose modifications are typical for ER resident glycoproteins, there are examples in the literature for cell-surface expressed, high mannose modified glycoproteins. However, the overall difference in protein modification of endogenous versus exogenous BST-2 could be indicative of differences in their relative intracellular distribution. This phenomenon needs to be taken into consideration in particular for future analyses of BST-2 intracellular trafficking.

## Competing interests

The authors declare that they have no competing interests financially or otherwise.

## Authors' contributions

AJA conceived the study, performed the molecular and biochemical studies, and assisted in writing the manuscript. EM performed biochemical and FACS analyses and helped with data analysis. SK assisted with BST-2 mutagenesis and biochemical analyses. KS coordinated and supervised the project and was involved in writing the manuscript.
